# Rehabilitation for a patient with anti-Yo antibody-positive paraneoplastic cerebellar degeneration caused by breast cancer: A case report and literature review

**DOI:** 10.1097/MD.0000000000008468

**Published:** 2017-11-03

**Authors:** Naoki Kato, Goichi Hashida, Kuni Konaka

**Affiliations:** a Department of Rehabilitation, Osaka University Medical Hospital; b Department of Neurology, Osaka University Graduate School of Medicine, Suita, Osaka, Japan.

**Keywords:** ataxia, breast neoplasms, paraneoplastic cerebellar degeneration, rehabilitation

## Abstract

**Rationale:**

Rehabilitation for paraneoplastic cerebellar degeneration (PCD) has been indicated as necessary. However, there are only a few reports on rehabilitation in PCD. We describe the course of physical and cognitive functions and activities of daily living (ADL) in a patient with PCD and examine the effect of rehabilitation, along with a review of relevant literature.

**Patient concerns:**

A 42-year-old woman experienced rapid deterioration in cerebellar symptoms and was unable to walk. The cerebellar symptoms improved after mastectomy, which was performed 3 months after the onset of symptoms. However, the cerebellar symptoms exacerbated 11 months after the onset of symptoms. She underwent immunotherapy because the level of anti-Yo antibodies was high, and anti-glutamic acid decarboxylase antibodies were detected.

**Diagnoses:**

She was diagnosed with anti-Yo antibody-positive PCD caused by breast cancer.

**Interventions:**

Rehabilitation was performed preoperatively, postoperatively, and at 1 year after the onset of symptoms.

**Outcomes:**

Her physical function and ADL improved after the surgery and at 1 year after the onset of symptoms. Finally, she regained the ability to walk between parallel bars supported with one hand and a walking frame.

**Lessons:**

Given the results of this case and the relevant literature, it appears that rehabilitation improves physical function and ADL after oncotherapy. Particularly, initiating treatment within three months of onset of symptoms may enable patients to walk without assistance.

## 1 Introduction

Paraneoplastic cerebellar degeneration (PCD) is a type of paraneoplastic neurological syndrome (PNS), and severe ataxia manifests over several days to weeks. PCD is associated with various cancers, such as lung, ovarian, and breast cancers. Antibody titers (such as anti-Yo, Hu, and Tr) are high, and they react with cerebellar Purkinje cells. The treatment includes oncotherapy and immunotherapy. Although patients are sometimes responsive to oncotherapy, immunotherapy is rarely successful, and the neurological prognosis is generally poor.^[1,2]^

Rehabilitation for PCD has been indicated as necessary^[1,2]^; however, there are only a few reports on rehabilitation in PCD.^[3–8]^ Furthermore, most reports only describe the changes in ADL after oncotherapy and immunotherapy. This report provides information regarding the course of physical and cognitive functions, and ADL (before and after surgery, and at 1 year after the onset of symptoms). It also examines the effects of rehabilitation in a patient with PCD along with a review of the relevant literature.

## 2 Case presentation

This study was approved by the Osaka University Medical Hospital ethical review board (Approval No. 13078), and signed consent was obtained from the patient's family.

A 42-year-old woman with an unremarkable medical history, family history, and psychosocial history experienced dizziness, which was a sustained feeling of floating and staggering and consulted a local physician. No neurological findings or abnormal findings on a magnetic resonance imaging scan of the head (head MRI) were observed.

She was admitted to the hospital because of a worsening of symptoms 2 months after their onset. She presented with cerebellar symptoms, including dizziness, trunk and limb ataxia, and dysarthria. She could walk between parallel bars supported by 1 hand and could care for self with supervision, but she required assistance while bathing. She had slightly impaired orientation. Miller Fisher syndrome was initially suspected and she received intravenous immunoglobulin (IVIg); however, her cerebellar symptoms continued to deteriorate rapidly. Subsequently, anti-Yo antibody was detected. After that, an abnormal accumulation in the left breast was found on positron emission tomography and it was diagnosed as left breast cancer (T1N0M0). Based on PNS diagnostic criteria in the European Federation of Neurological Societies, it was diagnosed as definite PCD. She underwent left mastectomy 1 month after admission. Histologically, the cancer was microinvasive ductal carcinoma; therefore, she received no postoperative treatment for the cancer, but she did receive IVIg and steroid pulse therapy for immunosuppression. Her cerebellar symptoms improved and she was transferred to a rehabilitation hospital 2 months after the surgery. At transfer, there were no abnormal findings on head MRI.

However, her cerebellar symptoms deteriorated again and cerebellar hemisphere atrophy was found on head MRI 4 months after the transfer. Seven months after the transfer, she was discharged, but was readmitted to the hospital for reexamination.

At readmission, there was no tumor recurrence or metastasis, but an analysis of her cerebrospinal fluid showed inflammation and high titers of anti-Yo antibodies. In addition, the level of anti-glutamic acid decarboxylase (GAD) antibody was elevated to 35.0 U/mL (standard value <5.0 U/mL). The level of anti-glutamic acid decarboxylase (GAD) antibody was elevated to 35.0 U/mL (standard value is <5.0 U/mL). She received IVIg, 2 courses of steroid pulse therapy, and plasma exchange (PE). The levels of inflammatory markers and anti-GAD antibodies improved, and the progression of cerebellar hemisphere atrophy was halted, but the cerebellar symptoms did not change. She was discharged 3 months after the readmission, and predonine and tacrolimus were prescribed for the suppression of recurrence.

Rehabilitation was performed during 3 periods: before and after the surgery, and at 1 year after the onset of symptoms. Before surgery, the rehabilitation program consisted of exercises for strengthening muscles, balance, gait between parallel bars, and ADL. Rehabilitation was performed for approximately 80 min/d, and the rehabilitation period was 2 weeks (every day on weekdays). After the surgery, the same rehabilitation program was followed for 10 weeks. At readmission, the same rehabilitation program was followed for 9 weeks (there was a temporary discharge for a total of 3 weeks).

The assessments of physical and cognitive functions and ADL are described in Table 1. During the first admission, her postoperative physical function and ADL had deteriorated compared with the initial assessments at admission; however, her muscle strength and cognitive function had improved slightly. She required both hands while walking between parallel bars and could not walk with a walking aid. In contrast, at transfer, her physical function, cognitive function, and ADL had improved compared with the postoperative assessments. She regained the ability to walk with the support of 1 hand between the parallel bars and a walking frame. During the readmission, at discharge, the Scale of the Assessment and Rating of Ataxia (SARA) score had not changed, but her muscle strength, balance, gait, and ADL had improved compared with the initial assessments at readmission. She was able to walk with the support of 1 hand between the parallel bars and a walking frame.

**Table 1:**
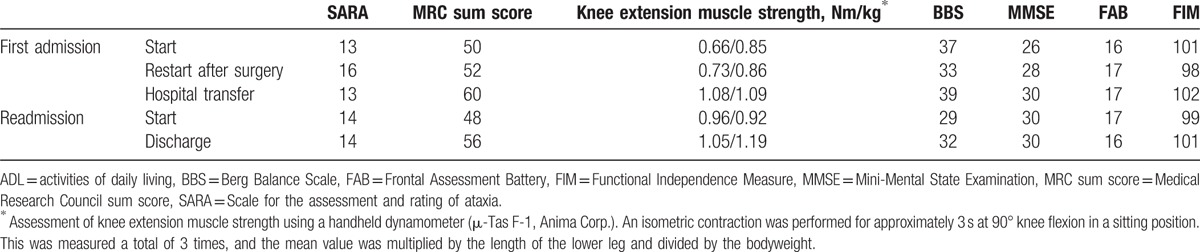
Physical function, cognitive function, and ADL.

Throughout these rehabilitative interventions, no adverse effects or unanticipated events occurred, and she continued rehabilitation with the goal of re-acquiring gait and ADL. Thus, she hoped to continue rehabilitation after discharge from our hospital, and she decided to continue outpatient rehabilitation at a local hospital.

## 3 Discussion

In the present case, cerebellar symptoms deteriorated rapidly before the surgery but improved after it. Early oncotherapy is essential for the successful treatment of PCD,^[1]^ as cerebellar plasticity may occur if treatment is started within 3 months after onset.^[9]^ Her surgery was performed 3 months after onset, thus enabling an improvement in cerebellar symptoms after the surgery. However, 8 months after the onset, the cerebellar symptoms deteriorated again, possibly due to cerebellar atrophy, which is sometimes found in PCD a few months after the onset.^[1,2]^ Alternatively, the possibility of anti-GAD antibody-associated cerebellar ataxia cannot be excluded. However, we thought that it was difficult to judge whether there was a strong association between cerebellar ataxia and anti-GAD antibodies. Thus, the exact cause of the renewed deterioration of cerebellar symptoms remains unclear. However, following immunotherapy the cerebellar atrophy did not advance, and the anti-GAD antibody levels improved as well.

The SARA score improved after the surgery but did not improve before the surgery or at 1 year after the onset. Rehabilitation for ataxia has been reported to improve SARA scores, balance, gait ability, and the Functional Independence Measure (FIM) score in spinocerebellar degeneration^[10]^; thus, rehabilitation is effective for indolent ataxia. In the present case, the progression of cerebellar symptoms might have outstripped the effects of rehabilitation before surgery and at 1 year after the onset of symptoms.

The Medical Research Council (MRC) sum score and knee extension muscle strength improved for all periods. Although she had participated in skiing competitions a few months before the onset, her knee extension muscle strength at the first admission was approximately half that for a healthy 40-year-old Japanese woman.^[11]^ Furthermore, at readmission, her muscle strength had deteriorated by 12 points in the MRC sum score and by 13% in knee extension muscle strength compared with that at transfer. Patients with ataxia present with muscle weakness due to reduced activity^[8,12]^; thus, her muscle weakness might have resulted from disuse because she rarely went out of her home. Therefore, the intensive rehabilitation during hospitalization improved her muscle strength, regardless of the cerebellar symptoms.

The Berg Balance Scale (BBS) score followed the same course as the SARA score did before and after the surgery but improved at 1 year after the onset of symptoms. Compared with the assessments at transfer, the BBS score had deteriorated greatly (10 points) while the SARA score had deteriorated slightly (1 point) at readmission. If the progression of cerebellar symptoms is gradual, balance improves with rehabilitation.^[10]^ In addition, balance requires not only coordination, but also a variety of other factors such as muscle strength, sensory function, and cognition, with a particularly profound effect on muscle strength.^[13,14]^ The BBS score might have deteriorated because of muscle weakness rather than ataxia; therefore, the score improved as muscle strength improved. Gait and FIM score followed the same course as the BBS score; thus, gait and ADL disabilities were mainly due to a balance disorder.

There have been reports of cases of PCD with cognitive dysfunction.^[15,16]^ In the present case, during the first admission, cognitive functions, as assessed by the Mini-Mental State Examination and Frontal Assessment Battery (FAB) scores, improved even before the surgery. The reason for this improvement is unclear, as there were no significant findings on head MRI. During readmission, the FAB score deteriorated slightly. As the cerebellum transmits output to the prefrontal cortex region, a relationship between the cerebellum and cognitive function, especially prefrontal functions, has been indicated.^[17,18]^ Head MRI at 8 months after the onset of symptoms indicated atrophy of the cerebellar hemisphere, which may have led to the deterioration of FAB score.

Using MEDLINE and Google Scholar, a literature review was conducted to locate reports on the effects of rehabilitation on physical function and ADL in PCD published between 1970 and 2016. A summary of these previous reports is provided in Table 2. All reports indicated a slight improvement in ADL, and in almost all reports^[3,4,7,8]^ rehabilitation was undertaken after oncotherapy. Only the patient in the report by Sliwa et al^[3]^ improved enough to be able to walk independently. That patient received oncotherapy within the 3-month window of cerebellar plasticity,^[9]^ while oncotherapy or PE was started >3 months after the onset of symptoms in the other previous cases where such information was provided. In the current case, surgery was performed 3 months after the onset of symptoms and the patient's balance, gait ability, and ADL improved after the surgery. Subsequently, the patient was able to walk between parallel bars and with a walking frame independently. Fewer than 15% of patients with PCD show improvements in cerebellar symptoms,^[19,20]^ and >75% with anti-Yo anti-body-positive PCD require wheelchairs to move around.^[20]^ However, rehabilitation appears to improve ADL after standard therapy (mainly oncotherapy), and, in particular, initiating the treatment within 3 months after the onset of symptoms may enable a patient to walk without assistance.

**Table 2:**
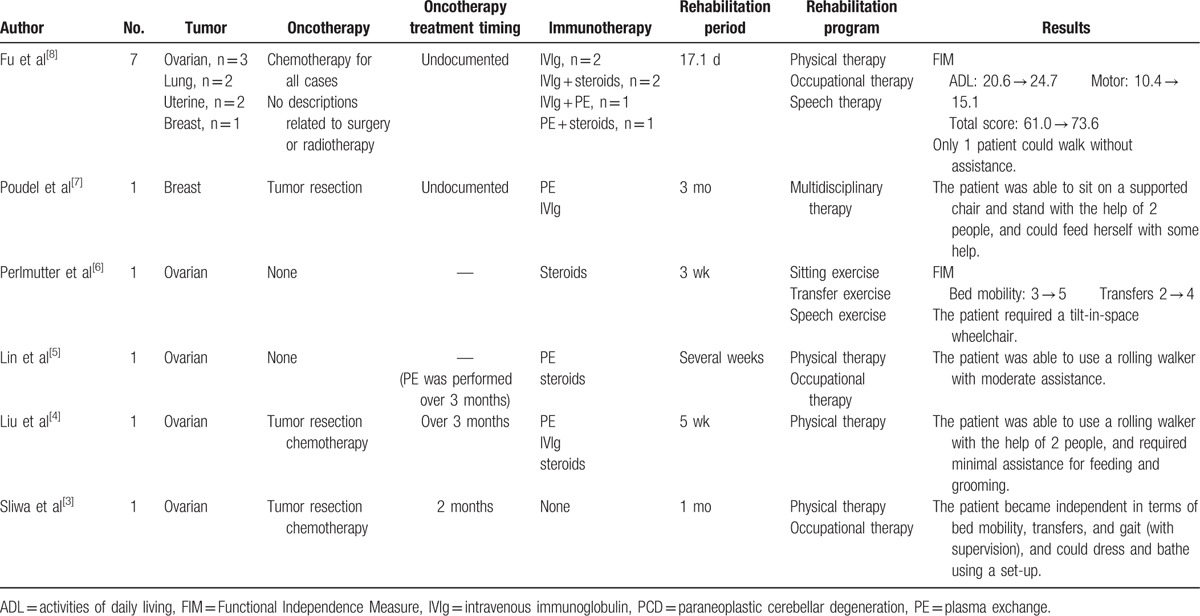
Reports on rehabilitation in PCD.

This study has some limitations. First, the evidence for the effectiveness of rehabilitation for PCD is limited because this is a case report. Second, the treatment at the time of first admission included mastectomy, IVIg, and steroid pulse. The treatment at the time of readmission included IVIg and steroid pulse; thus, we could not completely exclude the influence of these medical and surgical treatments on the improvement in physical and cognitive functions and ADL. However, muscle strength improved after the first admission and readmission, and BBS and FIM improved despite no changes in SARA during readmission. We believe that those were the results of rehabilitation.

In conclusion, physical function and ADL can improve with rehabilitation in patients with PCD when the progression of cerebellar symptoms is halted or improved. In addition, muscle strength can improve independent of the course of cerebellar symptoms. Therefore, in conjunction with early oncotherapy, it is important to continue rehabilitation according to the course of cerebellar symptoms. In the current case, improvement in cognitive function was also observed, which might have resulted due to a synergistic effect with rehabilitation. However, declines in cognitive function have been indicated; thus, awareness of this issue while implementing rehabilitation is also important.

## Acknowledgments

We would like to thank Editage (www.editage.jp) for English language editing.
